# Inhibition of GABAergic Neurotransmission by HIV-1 Tat and Opioid Treatment in the Striatum Involves μ-Opioid Receptors

**DOI:** 10.3389/fnins.2016.00497

**Published:** 2016-11-08

**Authors:** Changqing Xu, Sylvia Fitting

**Affiliations:** Department of Psychology and Neuroscience, University of North Carolina Chapel HillChapel Hill, NC, USA

**Keywords:** HIV-1 Tat, morphine, CTAP, μ-opioid receptor, GABA neurotransmission, striatum

## Abstract

Due to combined antiretroviral therapy (cART), human immunodeficiency virus type 1 (HIV-1) is considered a chronic disease with high prevalence of mild forms of neurocognitive impairments, also referred to as HIV-associated neurocognitive disorders (HAND). Although opiate drug use can exacerbate HIV-1 Tat-induced neuronal damage, it remains unknown how and to what extent opioids interact with Tat on the GABAergic system. We conducted whole-cell recordings in mouse striatal slices and examined the effects of HIV-1 Tat in the presence and absence of morphine (1 μM) and damgo (1 μM) on GABAergic neurotransmission. Results indicated a decrease in the frequency and amplitude of spontaneous inhibitory postsynaptic currents (sIPSCs) and miniature IPSCs (mIPSCs) by Tat (5–50 nM) in a concentration-dependent manner. The significant Tat-induced decrease in IPSCs was abolished when removing extracellular and/or intracellular calcium. Treatment with morphine or damgo alone significantly decreased the frequency, but not amplitude of IPSCs. Interestingly, morphine but not damgo indicated an additional downregulation of the mean frequency of mIPSCs in combination with Tat. Pretreatment with naloxone (1 μM) and CTAP (1 μM) prevented the Tat-induced decrease in sIPSCs frequency but only naloxone prevented the combined Tat and morphine effect on mIPSCs frequency. Results indicate a Tat- or opioid-induced decrease in GABAergic neurotransmission via μ-opioid receptors with combined Tat and morphine effects involving additional opioid receptor-related mechanisms. Exploring the interactions between Tat and opioids on the GABAergic system may help to guide future research on HAND in the context of opiate drug use.

## Introduction

Even though severe cases of human immunodeficiency virus type 1 (HIV-1)-associated dementia have been reduced due to the introduction of combined antiretroviral therapy (cART), 30–50%-infected individuals suffer from HIV-1-associated neurocognitive disorders (HAND) (Bell et al., 1998; Dore et al., 1999; Sacktor et al., 2002; Antinori et al., 2007; Ellis et al., 2007; Heaton et al., 2011; Saylor et al., 2016). HAND is specifically enhanced in the context of drug use, such as opiates, with worsening the consequences of HIV-1 in the central nervous system (CNS) (Bell et al., 1998; Arango et al., 2004; Anthony et al., 2005; Lan et al., 2015). Experimental models of acquired immune deficiency syndrome (AIDS) have further indicated an increase in virus and disease progression by opiates (Pérez-Casanova et al., 2007; Noel et al., 2008; Rivera-Amill et al., 2010; Bokhari et al., 2011; Hu et al., 2012; Masvekar et al., 2015). HIV-1 specifically targets subcortical and cortical areas of the brain, with HIV-1 positive individuals displaying deficits in sensorimotor function, executive function, learning and memory, and attention (Berger and Nath, 1997; Berger and Arendt, 2000; Heaton et al., 2011; Scott et al., 2011). The striatum is one of the regions with the highest viral burden (Kure et al., 1990; Berger and Arendt, 2000; Kumar et al., 2007) and rich in opioid receptor-expressing cells. Animal *in vivo* and *in vitro* studies focusing on the striatum have identified exacerbated behavioral and structural changes by opioids in the presence of the HIV-1 transactivator of transcription (Tat) corresponding to a HAND-associated phenotype (Fitting et al., 2010, 2012, 2014).

It has been demonstrated that Tat interacts with different receptors, including chemokine, NMDA, and G protein coupled receptors (Albini et al., 1998; Haughey et al., 2001; Prendergast et al., 2002; Eugenin et al., 2003; Feligioni et al., 2003; Longordo et al., 2006; Shin et al., 2012) leading to deficits in neurotransmission (Sabatier et al., 1991; Behnisch et al., 2004; Brailoiu et al., 2008; Hargus and Thayer, 2013), excitotoxic events (Haughey et al., 1999; Feligioni et al., 2003), synaptodendritic injury (Bertrand et al., 2014), and eventually neuronal death. Although synergism has been demonstrated between opioids and Tat on the glutamatergic system (Gurwell et al., 2001; Gupta et al., 2010; Zou et al., 2011; Fitting et al., 2014; Liu et al., 2016), not much is known about how HIV-1 Tat interacts with opioids on the GABAergic neurotransmitter system and whether the exacerbating effects of opioids in the presence of Tat can also be attributed to its effects on the GABAergic system. It is well known that opioids inhibit GABAergic neurotransmission in the striatum (Johnson and North, 1992; Klitenick et al., 1992; Bergevin et al., 2002) and recently the GABAergic inhibitory system has also been implicated in neuroAIDS, with the GABAergic inhibitory system being downregulated in HIV-1-positive patients with HAND (Gelman et al., 2012; Buzhdygan et al., 2016). In experimental studies it is less clear as Tat has been reported to increase inhibitory synapses and neurotransmission (Brailoiu et al., 2008; Hargus and Thayer, 2013), whereas other studies have reported unchanged or decreased inhibitory synapses or inhibitory release (Musante et al., 2010; Zucchini et al., 2013; Xu et al., 2016).

Thus, the present study investigated the effects of HIV-1 Tat alone and in combination with opioids by focusing on the GABAergic system. Results indicated a Tat and opioid-induced decrease in GABAergic neurotransmission via μ-opioid receptors with the combined effects of Tat and morphine involving more than just μ-opioid receptor-related mechanisms. Exploring the interactions between Tat and opioids on multiple neurotransmitter systems is important in understanding its effects on neuronal function and neuronal network processing.

## Materials and methods

### Electrophysiology

#### Slice preparation

All animal experiments were performed according to protocols approved by the Animal Care and Use Committee the University of North Carolina at Chapel Hill. Striatal slices were prepared from 14 to 24-day-old male and female C57BL/6J mice (The Jackson Laboratory, Bar Harbor, ME). Mice were euthanized using isoflurane, decapitated, and brains were placed into ice-cold sucrose buffer containing (in mM): 254 sucrose, 10 D-glucose, 26 NaHCO_3_, 2 CaCl_2_, 2 MgSO_4_, 3 KCl, and 1.25 NaH_2_PO_4_, saturated with 95% O_2_/5% CO_2_, at pH 7.4, 300 mOsm. Brains were cut into 300 μM coronal sections through the striatum using a VT 1000S microtome (Leica, Deerfield, IL) and placed into a holding chamber at 33°C for 30 min in a mixture of 50% sucrose saline and 50% artificial cerebrospinal fluid (aCSF) containing (in mM): 128 NaCl, 10 D-glucose, 26 NaHCO_3_, 2 CaCl_2_, 2 MgSO_4_, 3 KCl, and 1.25 NaH_2_PO_4_. Slices were then maintained at room temperature in aCSF bubbled continuously with 95% O_2_/5% CO_2_.

#### Recordings and analyses

Slices were transferred to a recording chamber (Warner Instruments, Hamden, CT) and continuously perfused with aCSF at 2–3 mL/min at ~33°C (Warner SC-20, Hamden, CT). Recordings were made in striatal medium spiny neurons (MSNs) located in the dorsolateral striatum that mainly integrate sensorimotor information (Pennartz et al., 2009) and are specifically affected in HIV-1 positive individuals (Bauer et al., 2005). Striatal MSNs were visualized using an Axio Examiner A1 microscope (Zeiss, Thornwood, NY) equipped with using differential interference contrast (DIC) and Dodt contrast. Patch-clamp recordings in whole-cell mode were obtained using a MultiClamp 700B amplifier (Axon Instruments, Union City, CA) and digitized using Digidata 1550A and pClamp 10.0 software (Molecular Devices, Sunnyvale, CA). Patch pipettes were glass capillaries with a filament (Narishige, Greencale, NY) pulled using a PC-10 puller (Narishige, Greencale, NY, #GD-1.2). The pipette internal solution used for whole-cell patch-clamp experiments consisted of (in mM) unless otherwise stated: 140 KCl, 0.1 CaCl_2_, 5 EGTA, 10 HEPES, 4 ATP-Mg^2+^, 0.4 GTP-2Na^+^, 1 QX314 (Lidocaine N-ethyl bromide), pH 7.2, 290 mOsm. The tip resistance of the patch electrode filled with internal solution was ~5 MΩ. Recordings were conducted immediately following treatment application and recording was conducted over a 5 min time period. Inhibitory postsynaptic currents (IPSCs) were recorded in the presence of the ionotropic glutamate receptor blockers 6,7-dinitroquinoxaline-2,3-dione (DNQX, 20 μM) and 2-amino-5-phosphopentanoate (AP-5; 20 μM), added to the superfusing aCSF. Spontaneous (s) and miniature (m) IPSCs were recorded at a holding potential of −70 mV and collected for 5 min for each treatment. The mIPSC recordings were obtained in the presence of 1 μM tetrodotoxin (TTX). Series resistance was monitored throughout the experiment. If the series resistance was unstable and changed >15% during the experiment, the cell was discarded. Signals were filtered at 2 kHz and digitized at 10 kHz. The Minianalysis software (Version 6.0.8; Synaptosoft, Decatur, GA) was used to perform off-line analysis.

### Treatments

HIV-1 Tat_1−86_ (5–50 nM, rtat HIV-1 IIIB, ImmunoDX, Woburn, MA), morphine sulfate (1 μM, NIDA Drug Supply System, Baltimore, MD) damgo (1 μM, Tocris, Ellisville, MO), naloxone (1 μM, Sigma-Aldrich, St. Louis, MO), and CTAP (D-Phe-Cys-Tyr-D-Trp-Arg-Thr-Pen-Thr-NH2; 1 μM, Tocris, Ellisville, MO) were dissolved in distilled water and administered by bath application. For the control we used bath application of distilled water by itself. As an additional control for Tat, we used heat-inactivated Tat_1−86_. Tat_1−86_ (50 nM) was heat-inactivated by incubation at 85°C for 30 min. Even though the concentration of Tat in the cerebral spinal fluid (CSF) has been reported at 1.14 nM (16 ng/mL) (Westendorp et al., 1995), the Tat concentration utilized in this study is generally accepted (Mayne et al., 2000; Haughey et al., 2001; Prendergast et al., 2002; Speth et al., 2002; Singh et al., 2004; Wallace et al., 2006; Brailoiu et al., 2008; Fitting et al., 2014). Opioid concentrations were based on previous studies (Johnson and North, 1992; Bergevin et al., 2002; McQuiston, 2007). DNQX (6,7-dinitroquinoxaline-2,3-dione, 20 μM), AP-5 (DL-2-amino-5-phosphonovaleric acid, 20 μM), and TTX (tetrodotoxin, 1 μM) were purchased from Tocris (Ellisville, MO). To manipulate levels we used three different experimental conditions: (1) aCSF without calcium, (2) aCSF with cadmium chloride (CdCl_2_, 200 μM, Sigma-Aldrich, St. Louis, MO), which blocks high and low threshold voltage-dependent calcium channels, and (3) endoplasmic reticulum calcium pump inhibitor thapsigargin (1 μM, Sigma-Aldrich, St. Louis, MO) that depletes the intracellular calcium stores.

### Statistics

All numerical values are expressed as mean ± standard error of the mean (SEM). Data were analyzed using paired Student's *t*-tests. An alpha level of *p* < 0.05 was considered significant.

## Results

### Tat concentration dependent effects on IPSCs in striatal slices

**Figure 1:**
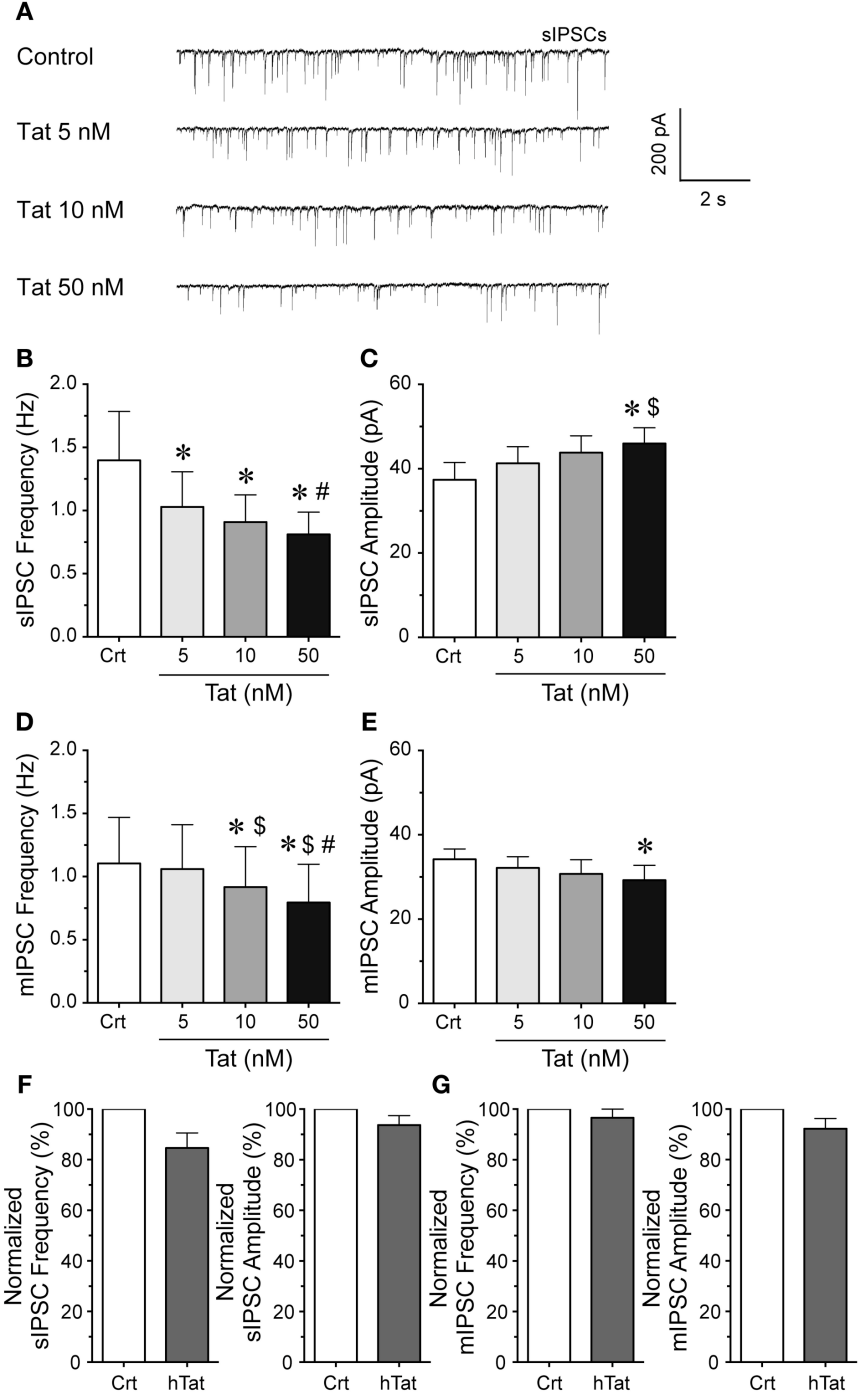
**Tat concentration-dependently decreased the frequency of sIPSCs and mIPSC in striatal MSNs. (A)** Representative traces show sIPSCs before and after application of Tat concentrations (5–50 nM). **(B)** Tat (5–50 nM) concentration-dependently decreased the mean frequency of sIPSCs (*n* = 13 neurons). **(C)** For the mean amplitude of sIPSCs only the highest Tat concentration (50 nM) increased the mean amplitude of sIPSCs (*n* = 13 neurons). **(D)** For mIPSCs, the mean frequency was concentration-dependently decreased by Tat (5–50 nM) (*n* = 15 neurons), whereas **(E)** the mean amplitude of mIPSCs showed no Tat effects except for a decrease by the highest Tat (50 nM) concentration (*n* = 15 neurons). **(F)** No effects for heat-inactivated Tat (50 nM) were noted on the normalized data of the mean frequency and mean amplitude of sIPSCs (*n* = 9 neurons). **(G)** No effects for heat-inactivated Tat (50 nM) were noted on the normalized data of the mean frequency and mean amplitude of mIPSCs (*n* = 10 neurons). Data are mean ± SEM. Significance was assessed by paired Student *t*-tests. ^*^p < 0.05 vs. Control,^#^*p* < 0.05 vs. Tat (10 nM), ^$^*p* < 0.05 vs. Tat (5 nM). Crt, Control; hTat, heat-inactivated Tat; MSNs, medium spiny neurons.

To explore the effects of Tat on GABergic neurotransmission patch-clamp recordings were performed on striatal MSNs (Figure 1). sIPSCs and mIPSCs were confirmed by the application of GABA_A_ receptor antagonist bicuculline (data not shown). The representative traces of sIPSCs before and after Tat application (5–50 nM) are shown in Figure 1A. As shown in Figure 1B, the mean (±SEM) frequency of sIPSCs (Hz) were as follows - for control: 1.34 ± 0.39, for Tat (5 nM): 1.03 ± 0.28, for Tat (10 nM): 0.91 ± 0.22, for Tat (50 nM): 0.81 ± 0.18; *n* = 13. A significant Tat effect was noted on sIPSCs, with all Tat concentrations demonstrating decreased mean frequency of sIPSCs compared to control [control vs. Tat (5 nM): *t*_(12)_ = 2.6, *p* = 0.023, control vs. Tat (10 nM): *t*_(12)_ = 2.5, *p* = 0.027, control vs. Tat (50 nM): *t*_(12)_ = 2.6, *p* = 0.023] and Tat (10 nM) significantly differing from Tat [50 nM; *t*_(12)_ = 2.3 *p* = 0.039]. As depicted in Figure 1C, the mean (±SEM) amplitude of sIPSCs (pA) were as follows - for control: 37.40 ± 4.09, for Tat (5 nM): 41.30 ± 3.94, for Tat (10 nM): 43.82 ± 4.00, for Tat (50 nM): 45.98 ± 3.74; *n* = 13. In contrast to mean sIPSC frequency, the mean amplitude of sIPSCs indicated only a significant increase by Tat (50 nM) compared to control [*t*_(12)_ = 2.7, *p* = 0.018] and compared to Tat (5 nM) [*t*_(12)_ = 2.5, *p* = 0.027]. To assess mIPSCs, TTX was added to the bath to eliminate large-amplitude, action potential-dependent IPSCs. As shown in Figure 1D, the mean (±SEM) frequency of mIPSCs (Hz) were as follows - for control: 1.10 ± 0.36, for Tat (5 nM): 1.06 ± 0.35, for Tat (10 nM): 0.92 ± 0.32, for Tat (50 nM): 0.79 ± 0.30; *n* = 15. A concentration dependent decrease in the frequency of mIPSCs by Tat was noted in the presence of TTX, with all conditions indicating significance from each other [control vs. Tat (10 nM): *t*_(14)_ = 2.7, *p* = 0.018, control vs. Tat (50 nM): *t*_(14)_ = 2.7, *p* = 0.016, Tat (5 nM) vs. Tat (10 nM): *t*_(14)_ = 2.3, *p* = 0.040, Tat (5 nM) vs. Tat (50 nM): *t*_(14)_ = 2.4, *p* = 0.031, Tat (10 nM) vs. Tat (50 nM): *t*_(14)_ = 2.3, *p* = 0.039], except for Tat (5 nM) compared to control. As depicted in Figure 1E, the mean (±SEM) amplitude of mIPSCs (pA) were as follows - for control: 34.18 ± 2.46, for Tat (5 nM): 32.14 ± 2.68, for Tat (10 nM): 30.72 ± 3.37, for Tat (50 nM): 29.23 ± 3.52; *n* = 15. The mean amplitude of mIPSCs indicated a significant decrease by Tat (50 nM) compared to control [*t*_(14)_ = 2.2, *p* = 0.049]. Heat-inactivated Tat (50 nM) revealed no significant effects on sIPSCs (*n* = 9 neurons, Figure 1F) and mIPSCs (*n* = 10 neurons, Figure 1G). Data were normalized to 100% for control and indicated following percent values for heat-inactivated Tat (50 nM): sIPSC frequency: 84.61 ± 5.97, sIPSC amplitude: 93.71 ± 3.73, mIPSC frequency: 96.54 ± 5.17, mIPSC amplitude 92.22 ± 4.04. Overall, Tat produced a significant decrease in action potential-dependent IPSCs (sIPSCs) and action potential-independent IPSCs (mIPSCs), with altering GABAergic neurotransmission presynaptically and partially postsynaptically, depending on Tat concentration. In the subsequent experiments Tat (10 nM) was used over Tat (50 nM) as 10 nM is closer to mimicking the physiological condition compared to 50 nM of Tat.

### Effects of morphine and damgo in combination with Tat

**Figure 2:**
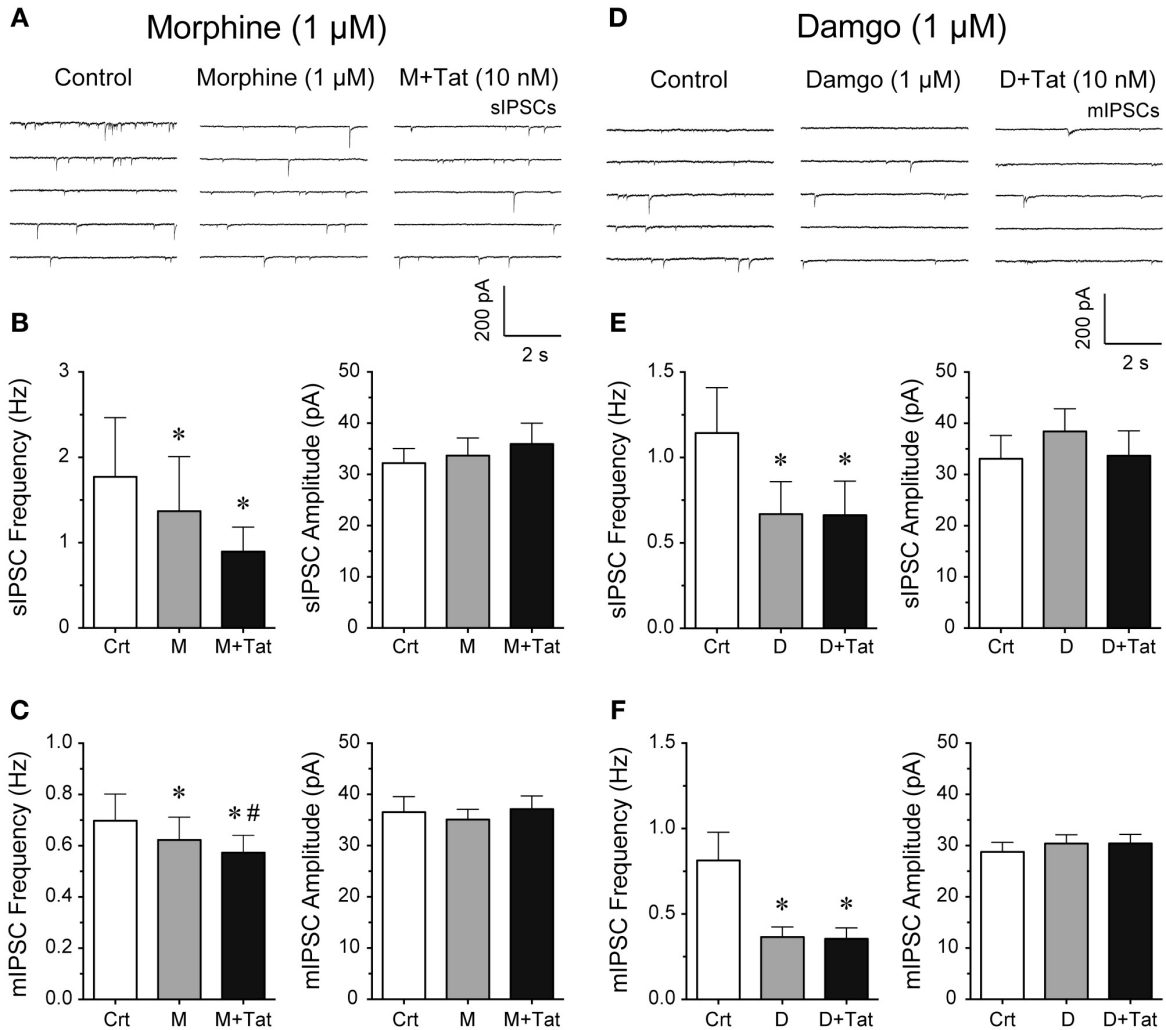
**Morphine and damgo significantly decreased the frequency of IPSCs with Tat inducing a further depression of mIPSCs only in combination with morphine. (A–C)** Morphine (1 μM) and Tat (10 nM) effects on IPSCs. **(A)** Representative traces show sIPSCs before morphine, after morphine, and after morphine + Tat application. **(B)** Morphine significantly decreased the mean frequency of sIPSCs, with morphine + Tat not changing the morphine-induced decrease in frequency of sIPSCs (*n* = 9 neurons). No effects were noted on the mean amplitude of sIPSCs (*n* = 9 neurons). **(C)** Morphine significantly decreased the mean frequency of mIPSCs with a further depression by Tat administration after morphine treatment (*n* = 7 neurons). No effects were noted on the mean amplitude of mIPSCs (*n* = 7 neurons). **(D–F)** Damgo (1 μM) and Tat (10 nM) effects on IPSCs. **(D)** Representative traces show mIPSCs before damgo, after damgo, and after damgo + Tat application. **(E)** Damgo significantly decreased the mean frequency of sIPSCs, with damgo + Tat not changing the damgo-induced decrease in frequency of sIPSCs (*n* = 9 neurons). No effects were noted on the mean amplitude of sIPSCs (*n* = 9 neurons). **(F)** Damgo significantly decreased the mean frequency of mIPSCs with no further depression of mIPSC frequency following Tat administration after damgo treatment (*n* = 11 neurons). No effects were noted on the mean amplitude of mIPSCs (*n* = 11 neurons). Data are mean ± SEM. Significance was assessed by paired Student *t*-tests. ^*^p < 0.05 vs. Control, ^#^*p* < 0.05 vs. M. Crt, Control; M, Morphine; D, Damgo.

It is known that acute bath application of opioids, such as morphine, decrease GABAergic neurotransmission in different brain regions (Bajo et al., 2014; Bobeck et al., 2014; Yousefpour et al., 2014), however, the combined effects of opioids and Tat on GABA release are less clear. Thus, we examined the effects of opioids and Tat on IPSCs by performing patch-clamp recordings on striatal MSNs with bath application of morphine (1 μM) or a highly selective μ-opioid agonist damgo (1 μM) followed by Tat (10 nM) administration (Figure 2). The representative traces of sIPSCs for control, morphine (1 μM), and morphine + Tat (10 nM) are shown in Figure 2A. As shown in Figure 2B (right Panel), the mean (±SEM) frequency of sIPSCs (Hz) were as follows - for control: 1.77 ± 0.69, for morphine (1 μM): 1.37 ± 0.64, for morphine + Tat (10 nM): 0.90 ± 0.29; *n* = 9. Morphine significantly reducing sIPSC frequency compared to control [*t*_(8)_ = 3.5, *p* = 0.008] with no further decrease by Tat (10 nM). As depicted in Figure 2B (left Panel), the mean (±SEM) amplitude of sIPSCs (pA) were as follows - for control: 32.20 ± 2.90, for morphine (1 μM): 33.68 ± 3.43, for morphine + Tat (10 nM): 35.93 ± 4.07; *n* = 9. No effects were noted on the mean amplitude of sIPSCs (Figure 2B). As shown in Figure 2C (right Panel), the mean (±SEM) frequency of mIPSCs (Hz) were as follows - for control: 0.70 ± 0.11, for morphine (1 μM): 0.61 ± 0.11, for morphine + Tat (10 nM): 0.57 ± 0.09; *n* = 7. For mIPSC frequency a significant morphine-induced decrease of mIPSC frequency was note compared to control [*t*_(6)_ = 3.3, *p* = 0.016, *n* = 7 neurons, Figure 2C], with being further downregulated by Tat [morphine (1 μM) vs. morphine + Tat (10 nM): *t*_(6)_ = 2.7, *p* = 0.034]. As depicted in Figure 2C (left Panel), the mean (±SEM) amplitude of mIPSCs (pA) were as follows - for control: 36.53 ± 3.04, for morphine (1 μM): 35.09 ± 2.01, for morphine + Tat (10 nM): 37.14 ± 2.55; *n* = 7. No effects were noted for the mean amplitude of mIPSCs (Figure 2C). As morphine acts on multiple subtypes of opioid receptors, we tested the selective μ-opioid receptor agonist damgo (1 μM). Similar effects were noted for damgo as seen with morphine. The representative traces of mIPSCs for control, damgo (1 μM), and damgo + Tat (10 nM) are shown in Figure 2D. As shown in Figure 2E (right Panel), the mean (±SEM) frequency of sIPSCs (Hz) were as follows - for control: 1.14 ± 0.27, for damgo (1 μM): 0.67 ± 0.19, for damgo + Tat (10 nM): 0.66 ± 0.20; *n* = 9. Damgo alone and damgo + Tat significantly decreased the mean frequency of sIPSC compared to control [*t*_(8)_ = 2.4, *p* = 0.045 and *t*_(8)_ = 2.4, *p* = 0.045, respectively]. As depicted in Figure 2E (left Panel), the mean (±SEM) amplitude of sIPSCs (pA) were as follows - for control: 33.08 ± 4.54, for damgo (1 μM): 38.40 ± 4.45, for damgo + Tat (10 nM): 33.69 ± 4.84, *n* = 9. No further downregulation of sIPSC frequency by Tat (10 nM) administration following damgo bath application was noted. As shown in Figure 2F (right Panel), the mean (±SEM) frequency of mIPSCs (Hz) were as follows - for control: 0.81 ± 0.16, for damgo (1 μM): 0.37 ± 0.06, for damgo + Tat (10 nM): 0.35 ± 0.06; *n* = 11. Similarly, damgo alone and damgo + Tat reduced the mean frequency of mIPSCs compared to control [*t*_(10)_ = 2.6, *p* = 0.027 and *t*_(10)_ = 2.7 *p* = 0.022, respectively]. As depicted in Figure 2F (left Panel), the mean (±SEM) amplitude of mIPSCs (pA) were as follows - for control: 28.77 ± 1.87, for damgo (1 μM): 30.41 ± 1.71, for damgo + Tat (10 nM): 30.42 ± 1.77, *n* = 11. No effects were noted on the mean amplitude of sIPSCs (Figure 2E) and mIPSCs (Figure 2F). Thus, morphine and damgo decrease GABAergic neurotransmission probably via a pre-synaptic mechanism as the mean amplitude of IPSCs was not significantly affected by opioids. Additionally, combined treatment of morphine and Tat further decreased action potential-independent GABA release presynaptically, which was not noted with combined damgo and Tat treatment.

### Combined Tat and morphine effects are blocked by naloxone and partially by CTAP

**Figure 3:**
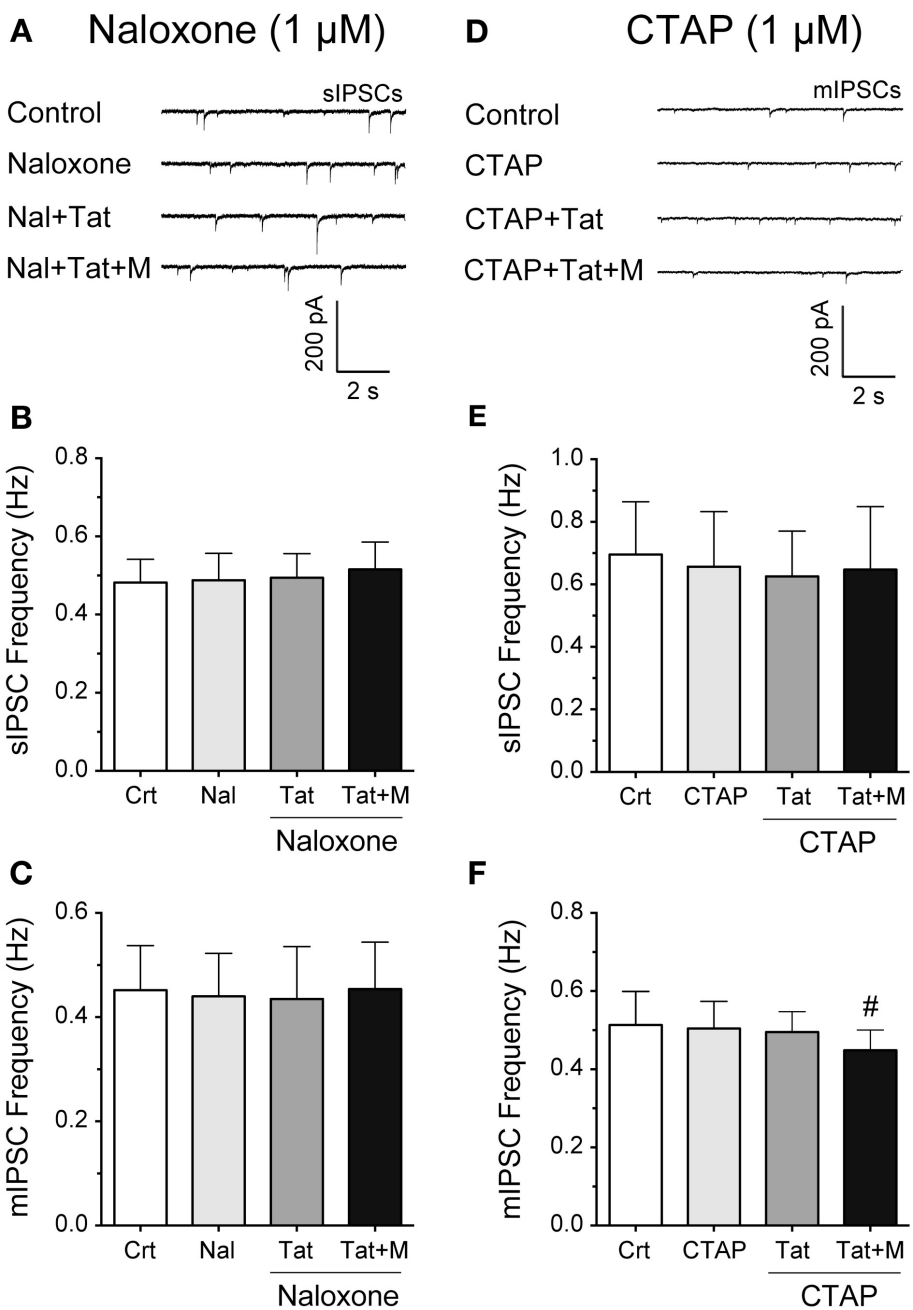
**Naloxone and partially CTAP blocked the effects of Tat and/or morphine on the frequency of IPSCs. (A–C)** Naloxone (1 μM), Tat (10 nM), and morphine (1 μM) effects on the mean frequency of IPSCs. **(A)** Representative traces show sIPSCs before and after naloxone treatment, followed by Tat, and morphine application. **(B)** No significant effects were noted on the mean frequency of sIPSCs for Tat or combined Tat and morphine treatment in the presence of naloxone (*n* = 12 neurons). **(C)** Similarly, in the presence of naloxone, no significant effects were noted on the mean frequency of mIPSCs for Tat (10 nM) or combined Tat and morphine treatment (*n* = 10 neurons). **(D–F)** CTAP (1 μM), Tat (10 nM) and morphine (1 μM) effects on the mean frequency of IPSCs. **(D)** Representative traces show mIPSCs before and after CTAP treatment, followed by Tat, and morphine (1 μM) application. **(E)** No significant effects were noted on the mean frequency of sIPSCs for Tat or combined Tat and morphine treatment in the presence of CTAP (*n* = 11 neurons). **(F)** In the presence of CTAP, combined Tat and morphine treatment significantly downregulated the mean frequency of mIPSCs compared to Tat alone (*n* = 10 neurons). Data are mean ± SEM. Significance was assessed by paired Student *t*-tests. ^#^*p* < 0.05 vs. CTAP + Tat. Crt, Control; M, Morphine.

To examine more in detail through which receptor the combined Tat and morphine-induced decrease in GABAergic neurotransmission is mediated, striatal slices were pretreated with naloxone (1 μM), a high affinity antagonist for the μ-opioid receptor but also some affinity for the κ-, and δ-opioid receptors, or CTAP (1 μM), a selective μ-opioid receptor antagonist (Figure 3). The representative traces of sIPSCs for naloxone (1 μM) followed by Tat (10 nM) and then morphine (1 μM) administration are shown in Figure 3A. As shown in Figure 3B, the mean (±SEM) frequency of sIPSCs (Hz) were as follows - for control: 0.48 ± 0.06, for naloxone (1 μM): 0.49 ± 0.07, for naloxone + Tat (10 nM): 0.49 ± 0.06, for naloxone + Tat + morphine (1 μM): 0.52 ± 0.07. Pretreating striatal slices with the opioid antagonist naloxone (1 μM) revealed no significant effects for Tat (10 nM) or combined Tat and morphine (1 μM) treatment on the mean frequency of sIPSCs (*n* = 12 neurons, Figure 3B) and the mean frequency of mIPSCs (*n* = 10 neurons, Figure 3C). As shown in Figure 3C, the mean (±SEM) frequency of mIPSCs (Hz) were as follows - for control: 0.45 ± 0.09, for naloxone (1 μM): 0.44 ± 0.08, for naloxone + Tat (10 nM): 0.44 ± 0.10, for naloxone + Tat + morphine (1 μM): 0.45 ± 0.09. No significant effects were noted on the mean amplitude of sIPSCs and mIPSCs (data not shown). To test if the combined Tat (10 nM) and morphine (1 μM) effects were μ-opioid receptor specific, CTAP (1 μM) was used as a pretreatment (Figures 3D–F). The representative traces of mIPSCs for CTAP (1 μM) followed by Tat (10 nM) and then morphine (1 μM) administration are shown in Figure 3D. As shown in Figure 3E, the mean (±SEM) frequency of sIPSCs (Hz) were as follows - for control: 0.70 ± 0.17, for CTAP (1 μM): 0.66 ± 0.18, for CTAP + Tat (10 nM): 0.63 ± 0.14, for CTAP + Tat + morphine (1 μM): 0.65 ± 0.20. Similar to naloxone, CTAP (1 μM) revealed no significant effects for Tat (10 nM) or combined Tat and morphine (1 μM) treatment on the mean frequency of sIPSCs (*n* = 11 neurons, Figure 3E). As shown in Figure 3F, the mean (±SEM) frequency of mIPSCs (Hz) were as follows - for control: 0.51 ± 0.07, for CTAP (1 μM): 0.50 ± 0.07, for CTAP + Tat (10 nM): 0.48 ± 0.07, for CTAP + Tat + morphine (1 μM): 0.44 ± 0.06. Interestingly, whereas CTAP (1 μM) blocked the significant effects of Tat (10 nM) on the mean frequency of sIPSCs, the significant effect of combined Tat and morphine (1 μM) treatment on mIPSC frequency was not blocked [CTAP (1 μM) + Tat (10 nM) vs. CTAP + Tat + morphine (1 μM) *t*_(9)_ = 3.0, *p* = 0.016, *n* = 10 neurons, Figure 3F]. No significant effects were noted on the mean amplitude of sIPSCs and mIPSCs (data not shown). Thus, the significant decrease of GABA release with combined Tat and morphine treatment appears to involve predominantly μ-opioid receptor-related mechanisms.

### Tat effects on IPSCs depend on extracellular and intracellular calcium

**Figure 4:**
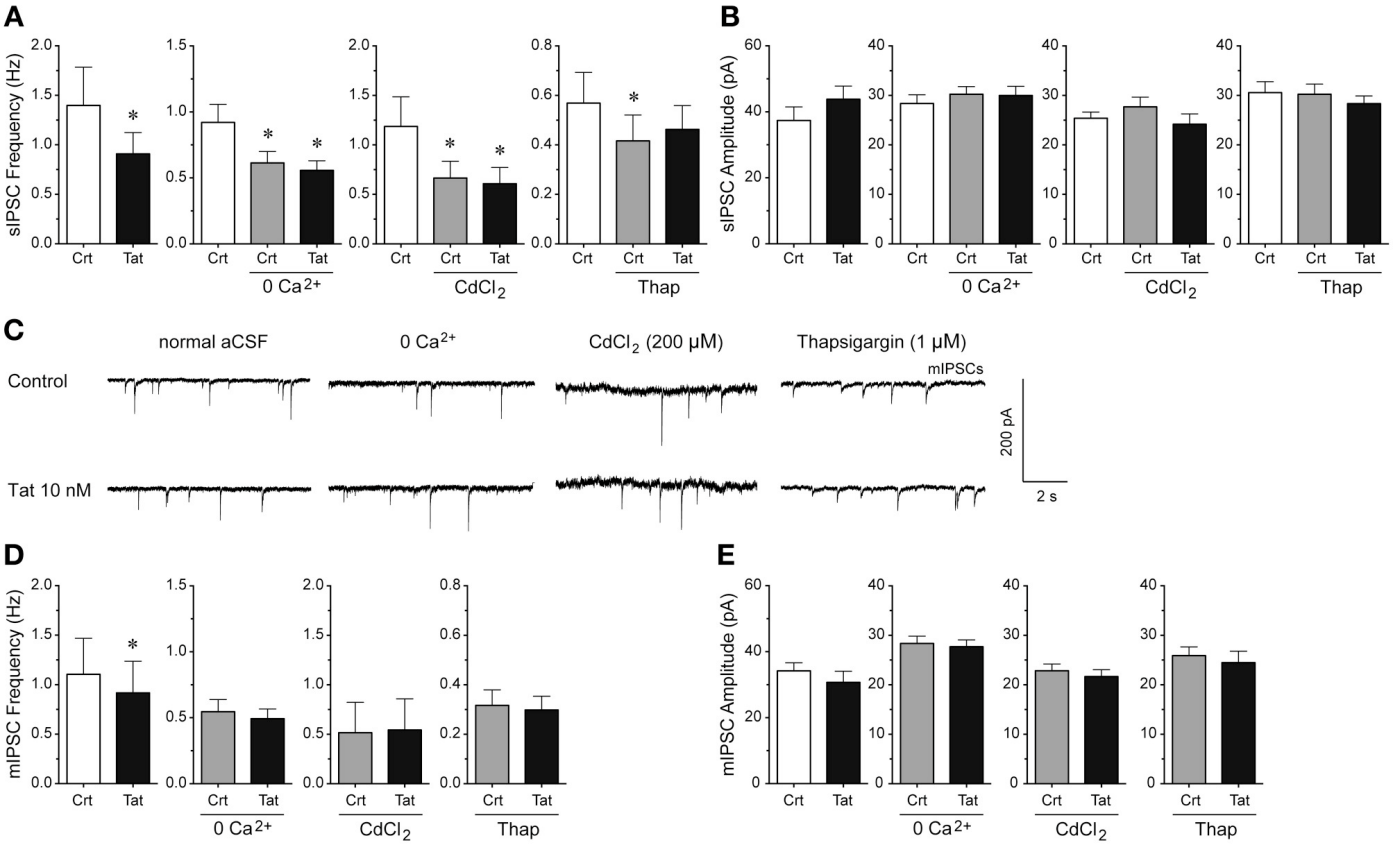
**Extracellular and intracellular calcium contribute to the significant Tat (10 nM) effects on the mean frequency of IPSCs. (A)** The significant Tat (10 nM) effect on the mean frequency of sIPSCs (*n* = 13 neurons) was blocked by zero extracellular Ca^2+^ (*n* = 13 neurons), CdCl_2_ (200 μM, *n* = 10 neurons), and thapsigargin (1 μM, *n* = 10 neurons). **(B)** No Tat effects were noted on the mean amplitude of sIPSCs. **(C)** Representative traces show sIPSCs before and after application of Tat (10 nM) in the presence of normal aCSF, zero extracellular Ca^2+^, CdCl_2_ (200 μM), and thapsigargin (1 μM). **(D)** The significant Tat (10 nM) effect on the mean frequency of mIPSCs (*n* = 15 neurons) was blocked by zero extracellular Ca^2+^ (*n* = 8 neurons), CdCl_2_ (200 μM, *n* = 9 neurons), and thapsigargin (1 μM, *n* = 10 neurons). **(E)** No Tat effects were noted on the mean amplitude of mIPSCs in any condition. Data are mean ± SEM. Significance was assessed by paired Student *t*-tests. ^*^p < 0.05 vs. Control of corresponding condition. aCSF, artificial cerebrospinal fluid; Crt, Control; Thap, Thapsigargin.

To understand the mechanisms by which Tat decreases GABAergic synaptic neurotransmission, we examined the involvement of extracellular calcium, voltage-gated calcium channels, and intracellular calcium (Figure 4). Manipulating extracellular and intracellular calcium decreased the frequency of sIPSCs by 33.3% when removing extracellular calcium from the aCSF [control vs. 0 Ca^2+^ control: *t*_(12)_ = 4.1, *p* < 0.001, *n* = 13 neurons], by 43.9% when blocking the voltage-gated calcium channels with CdCl_2_ [200 μM; control vs. CdCl_2_ control: *t*_(9)_ = 3.5, *p* = 0.006, *n* = 10 neurons], and by 26.9% when depleting intracellular calcium stores with the endoplasmic reticulum calcium pump inhibitor thapsigargin [control vs. thapsigargin control: *t*_(9)_ = 2.3, *p* = 0.050, *n* = 10 neurons; Figure 4A]. Importantly, whereas Tat (10 nM; 0.91 ± 0.22) significantly reduced the frequency of sIPSCs in the presence of normal aCSF (1.34 ± 0.39) [*t*_(12)_ = 2.5, *p* = 0.027, *n* = 13 neurons], no Tat effects were noted when extracellular calcium was removed from the aCSF [*n* = 13 neurons; control: 0.61 ± 0.09, Tat (10 nM): 0.56 ± 0.07], voltage-gated calcium channels were blocked with CdCl_2_ [200 μM; *n* = 10 neurons; control: 0.67 ± 0.17, Tat (10 nM): 0.61 ± 0.16], or intracellular calcium stores were depleted from calcium with thapsigargin [1 μM; *n* = 9 neurons; control: 0.42 ± 0.10, Tat (10 nM): 0.46 ± 0.10; Figure 4A], indicating that extracellular and intracellular calcium are necessary for the Tat-induced decrease in sIPSC frequency. No significant overall treatment effects were noted for the amplitude of sIPSCs for any condition (Figure 4B). As shown in Figure 4B, the mean (±SEM) amplitude of sIPSCs (pA) for the different conditions were as follows—control condition: normal aCSF: 37.40 ± 4.09, Tat (10 nM): 43.82 ± 4.00; zero extracellular Ca^2+^: aCSF: 30.24 ± 1.55, Tat (10 nM): 29.99 ± 1.85; CdCl_2_: aCSF: 27.69 ± 1.97, Tat (10 nM): 24.16 ± 2.10; thapsigargin: aCSF: 30.24 ± 2.05, Tat (10 nM): 28.35 ± 1.55. For mIPSCs, the representative traces before and after Tat application for each condition are shown in Figure 4C. Similar to effects on sIPSCs, Tat (10 nM; 0.92 ± 0.32) significantly downregulated the frequency of mIPSCs in normal aCSF (1.10 ± 0.36) [*t*_(14)_ = 2.7, *p* = 0.018, *n* = 15 neurons], but no significant Tat effects were noted in the absence of extracellular calcium [*n* = 8 neurons; control: 0.55 ± 0.09, Tat (10 nM): 0.49 ± 0.07], in the presence of CdCl_2_ [200 μM; *n* = 9 neurons; control: 0.50 ± 0.31, Tat (10 nM): 0.54 ± 0.31], and in the presence of thapsigargin [1 μM; *n* = 10 neurons; control: 0.32 ± 0.06, Tat (10 nM): 0.30 ± 0.06; Figure 4D]. Further, no significant Tat (10 nM) effects were noted for the amplitude of mIPSCs (Figure 4E). As shown in Figure 4E, the mean (±SEM) amplitude of mIPSCs (pA) for the different conditions were as follows—control condition: normal aCSF: 34.18 ± 2.46, Tat (10 nM): 30.72 ± 3.37; zero extracellular Ca^2+^: aCSF: 28.36 ± 1.46, Tat (10 nM): 27.69 ± 1.39; CdCl_2_: aCSF: 22.83 ± 1.37, Tat (10 nM): 21.66 ± 1.39; thapsigargin: aCSF: 25.90 ± 1.76, Tat (10 nM): 24.47 ± 2.32. Thus, the significant Tat (10 nM) effects on the frequency of IPSCs were abolished when removing extracellular and/or intracellular calcium, indicating that extracellular and intracellular calcium are necessary for the Tat-induced decrease in GABA release.

## Discussion

In the present study, we investigated (1) the HIV-1 Tat effects on the GABAergic neurotransmitter system, (2) the combined effects of Tat and opioids on the GABAergic system, and (3) the underlying mechanisms by which Tat affects GABAergic neurotransmission.

### Tat concentration-dependently decreases GABAergic neurotransmission

It is well known that Tat is excitotoxic by activating glutamatergic NMDA receptors (Magnuson et al., 1995; Haughey et al., 2001; Pérez et al., 2001; Longordo et al., 2006; Li et al., 2008; Aksenov et al., 2012). Further, Tat has been shown to directly affect glutamatergic neurotransmission by increasing the frequency of miniature excitatory postsynaptic currents (mEPSCs) (Brailoiu et al., 2008) and neuronal excitability (Ngwainmbi et al., 2014; Fitting et al., 2015). However, as glutamate and GABA are the most abundant neurotransmitters in the brain, neuronal excitability is critically dependent on the level of inhibition, and accordingly changes of inhibitory synaptic efficacy has great impact on neuronal function and neuronal network processing (Petroff, 2002).

The present study provides direct evidence that Tat concentration-dependently decreases inhibitory transmitter release in the dorsolateral striatum. The observation that Tat alters sIPSCs and mIPSCs in medium spiny GABAergic projection neurons (MSNs) indicates that Tat has effects on intrinsic interneuron excitability and modifies the GABA release machinery. Specifically, our data suggest that action potential (AP)-independent IPSCs (mIPSCs) contribute mostly to overall IPSCs in our MSN recordings. It is known that AP-independent spontaneous vesicular release of GABA in the CNS mediates mIPSCs (Axmacher et al., 2004). Another source of mIPSCs is known to be the reversal of GABA transporter (GAT), which may result in additional GABA release under certain physiological conditions (Allen et al., 2004; Wu et al., 2007; Jin et al., 2011). Further, as in the present study glutamate receptor blockers were present in the bath, the GABA component of the AP-dependent synaptic potential, which arises largely from collateral branches of the MSNs themselves, was inhibited, thus contributing to smaller AP-dependent GABA release (sIPSCs). The marked reduction of the mean frequency of IPSCs by Tat (10 nM), without any change in the mean amplitude of IPSCs, suggests that Tat acts at presynaptic GABAergic terminals and suppresses inhibitory synaptic input to MSNs. As a decrease in inhibitory synaptic inputs would facilitate an increase in postsynaptic activity, the presynaptic inhibition of IPSCs may play a role in the excitatory effects of Tat on the activities of MSNs, besides direct excitation through postsynaptic actions (Kim et al., 2008).

Interestingly, in the present study, the highest concentration of Tat (50 nM) altered the amplitude of IPSCs, suggesting that Tat may modulate IPSCs also through postsynaptic mechanisms, such as alterations in number of postsynaptic receptors, postsynaptic sensitivity or conductance. The discrepancy of Tat (50 nM) increasing the amplitude of sIPSCs but decreasing the amplitude of mIPSCs is not well understood. For the increased Tat effects noted on the sIPSC amplitude, AP-driven events may play a role due to intrinsic properties of the presynaptic cell or network activity. It is suggested that tonically active GABAergic interneurons might contribute to the Tat-induced increase in sIPSC amplitude as fast-spiking parvalbumin interneurons in the pyramidal layer have been demonstrated to be selectively vulnerable to Tat (Marks et al., 2016). Additionally, other types of GABAergic interneurons make connections with the spiny neurons, including interneurons that express tyrosine hydroxylase (Xenias et al., 2015) and neuropeptide Y (Beatty et al., 2012). As we did notice a decrease in sIPSC frequency, it should be noted that the probability of opening also depends upon how much GABA is released (presynaptic), which influences the sIPSC frequency. In contrast, mIPSC amplitude is AP-independent, thus Tat effects on postsynaptic receptors, such as single channel conductance through an open GABA pore, or a change in the number of channels could contribute to a postsynaptic decrease in mIPSC amplitude. Further, the decrease in mIPSC amplitude could be due to the internalization of specific receptors. TNF-α is known to causes internalization of GABA_A_ receptors, resulting in fewer surface GABA_A_ receptors and a decrease in inhibitory synaptic strength (Stellwagen et al., 2005). TNF-α has been shown to play a central role in initiating inflammatory cascades in Tat-exposed astroglia and microglia (Benveniste and Benos, 1995; Luo et al., 2003; El-Hage et al., 2008) as well as in HIV-infected patients (Zhao et al., 2014). It is important to note however, that the effect on amplitude was only noticed for the high Tat (50 nM) concentration, in contrast to Tat (10 nM) that did not produce significant effects on ISPC amplitude.

Further, the effects of Tat on the inhibitory GABAergic system indicate to be variable with multiple studies reporting different results (Brailoiu et al., 2008; Fitting et al., 2013; Hargus and Thayer, 2013; Zucchini et al., 2013; Marks et al., 2016). A recent study demonstrated that Tat applied *in vitro* as well as expressed in the brain *in vivo* induced a decrease in GABA exocytosis in the cortex, while GABA exocytosis was unchanged in the hippocampus (Zucchini et al., 2013). Further, it appears that Tat has various effects on different inhibitory synaptic proteins, with dowregulating synaptotagmin 2 (Syt2) in the hippocampus of Tat transgenic mice (Fitting et al., 2013), whereas upregulating gephyrin in the hippocampus *in vitro* and *in vivo* (Fitting et al., 2013; Hargus and Thayer, 2013). Thus, depending on the brain region and the inhibitory synaptic input the GABAergic system might be differently affected by Tat.

Overall the present study demonstrates that Tat modulates inhibitory GABAergic influence on MSNs in the dorsolateral striatum through both presynaptic and postsynaptic mechanisms, depending on Tat concentration.

### Differential effects of Tat in combination with morphine or damgo on the GABAergic system

Multiple studies have investigated the effects of opioids on GABAergic synaptic transmission with demonstrating that opioids inhibit GABA-mediated neurotransmission (Vaughan and Christie, 1997; Vaughan et al., 1997; Zhang et al., 2015). As reported in the present study it has been shown previously that opioids decrease the frequency of IPSCs but do not change the amplitude of IPSCs, indicating that neurotransmission is inhibited presynaptically (Vaughan and Christie, 1997; Vaughan et al., 1997). More interestingly, whereas a combined effect was noted for morphine and Tat on the frequency of mIPSCs, the selective μ-opioid receptor agonist damgo did not further downregulate mIPSC frequency in the presence of Tat. It is known that morphine and damgo, which primarily act on the μ-opioid receptors, have high efficacy in inhibiting calcium channels, whereas damgo but not morphine has high efficacy for p38 mitogen-activated protein kinase activation that regulates inflammation and is activated by cytokine production (Tibbles and Woodgett, 1999; Tan et al., 2009). As Tat has been shown to activate p38 mitogen-activated protein kinase (Li et al., 2005; Li and Lau, 2007; Gupta et al., 2010), differential caspase-dependent pathways could be used by Tat and morphine to inhibit GABA release and cause a downregulation when applied in combination.

Additionally, in contrast to damgo, morphine is not selective to μ-opioid receptors but also has effects on other opioid receptors. That other subtypes of opioid receptors besides the μ-opioid receptor seem to be involved in the combined opioid and Tat effects on GABAergic neurotransmission is supported by the finding that naloxone but not CTAP was able to block the combined opioid and Tat effects on GABAergic neurotransmission, specifically for mIPSC frequency. It should be noted that the μ-opioid receptor inhibition with CTAP blocked combined Tat and morphine-induced decrease in sIPSC frequency, but not in mIPSC frequency. The involvement of δ-opioid receptors has been shown in a previous study, indicating that a selective δ-opioid receptor agonist (D-Pen(2,5)-enkephalin, DPDPE) but not a selective κ-opioid receptors agonist [(+)-(5 alpha,7 alpha,8 beta)-N-methyl-N-[7-(1-pyrrolidinyl)-1-oxaspiro[4.5]dec-8-yl]-benzeneacetamide; U-69593] decreased IPSCs with a co-localization of δ-opioid receptors and dopamine 1 (D_1_)-type receptors in the rat dorsolateral striatum (Ambrose et al., 2006). Naloxone is a high affinity antagonist for the μ-opioid receptor but also has some affinity on the κ-, and δ-opioid receptors, whereas CTAP is a selective μ-opioid receptor antagonist. Previous studies have shown that κ-, and δ-opioid receptors are involved in the regulation of inhibitory transmission (Ford et al., 2006; Bosse et al., 2014; Gilpin et al., 2014). Specifically, δ-opioid receptors inhibit GABA release via a presynaptic site of action in the striatum (Jiang and North, 1992) and are localized mainly to presynaptic terminals of inhibitory synapses (Svingos et al., 1998). In addition to a regulating function, δ-opioid receptors have also been shown to downregulate GABA_A_ receptor expression in the cortex of δ-opioid receptor transgenic mice (Feng et al., 2011). Besides morphine's effects on μ- opioid receptors but also on κ-, and δ-opioid receptors, it additionally has been shown that morphine can activate nociceptin/orphanin FQ peptide receptors (Ueda et al., 2000; Kest et al., 2001). The specific receptor that might contribute to the further downregulation of GABA release by combined Tat and morphine treatment can not be conclusively determined in the present study and needs further investigation.

Additionally, it needs to be pointed out that certain subpopulation of MSNs might show differential responsiveness to treatments, such as Tat and opioids. μ-opioid receptors are generally thought to reside in striosome (not matrix) MSNs (Cui et al., 2014). Even though μ-opioid receptors are expressed in both the D_1_-type MSNs of the direct pathway and D_2_-type MSNs of the indirect pathway, it appears that at least in some striosomes there is an overabundance of D_1_-type MSNs (Fujiyama et al., 2011; Watabe-Uchida et al., 2012; Cui et al., 2014), and further μ-opioid receptors might be specifically located in D_1_-MSNs of the direct pathway (Gerfen, 1985; Crittenden and Graybiel, 2011; Cui et al., 2014). Thus, the differential contribution of the D_1_-type MSNs and D_2_-type MSNs to the effects of morphine and damgo in the context of Tat exposure needs to be assessed in more detail.

### Decrease of GABAergic neurotransmission by Tat is dependent on extracellular and intracellular calcium

Studies have demonstrated that Tat alters calcium with inducing abnormal and excessive calcium influx and increasing intracellular calcium release that consequentially elevates cytosolic free calcium levels and leads to neurotoxicity (Haughey and Mattson, 2002; Hu, 2016). However, little research has been done to examine the involvement of calcium in the context of Tat on GABAergic synaptic neurotransmission. GABA has been shown to be released via calcium-dependent mechanisms that involve different subtypes of voltage-gated calcium channels (Alamilla and Gillespie, 2013; Nelson et al., 2014). The present study demonstrates that the observed Tat-induced decrease in GABAergic neurotransmission is dependent on extracellular and intracellular calcium. It is known that Tat increases calcium via inositol 1,4,5-trisphosphate (IP_3_)-regulated and/or ryanodine-regulated pools (Lipton, 1994; Nath and Geiger, 1998; Haughey et al., 1999; Fitting et al., 2014) with subsequent increases in calcium that enters the cell extracellularly via voltage-gated calcium channels (Bonavia et al., 2001). An increase in intracellular calcium could thus, account for the reduction in the amplitude of mIPSCs seen in the present study, as increases in intracellular calcium can lead to a suppression of the mean amplitude of IPSCs (Brussaard et al., 1996). Interestingly, there was an increase noted for the mean amplitude of sIPSCs by Tat (50 nM), indicating that the AP-dependent release of GABA from transmitter vesicles was decreased for frequency but increased for amplitude. As the increase in amplitude was only seen for sIPSCs and not mIPSCs the amplitude change could be related to a selective effect on intrinsic interneuron excitability and needs to be further studied.

The present study further suggests that the effects of Tat on GABA release appear to be mediated via μ-opioid receptors, as CTAP was able to block the Tat-induced decrease of GABA release. Opioid receptors are widely expressed in the striatum and the GABA modulation by μ-opioid receptors is consistent with anatomical data showing that neurons are immunopositive for μ-opioid receptors on pre- (axonal) and postsynaptic (dendritic) locations in different brain regions (Arvidsson et al., 1995; Drake and Milner, 1999). Additionally, it is known that μ-opioid receptors are extensively co-localized with paravalbumin-positive neurons (Drake and Milner, 2006), with a recent study demonstrating that neurons expressing parvalbumin in the pyramidal layer and neurons expressing somatostatin in stratum oriens are selectively vulnerable to Tat (Marks et al., 2016). Overall, the present study demonstrates that the Tat-induced decrease in GABAergic neurotransmission is dependent on extracellular and intracellular calcium, potentially involving a μ-opioid receptor-related pathway.

## Conclusions

**Figure 5:**
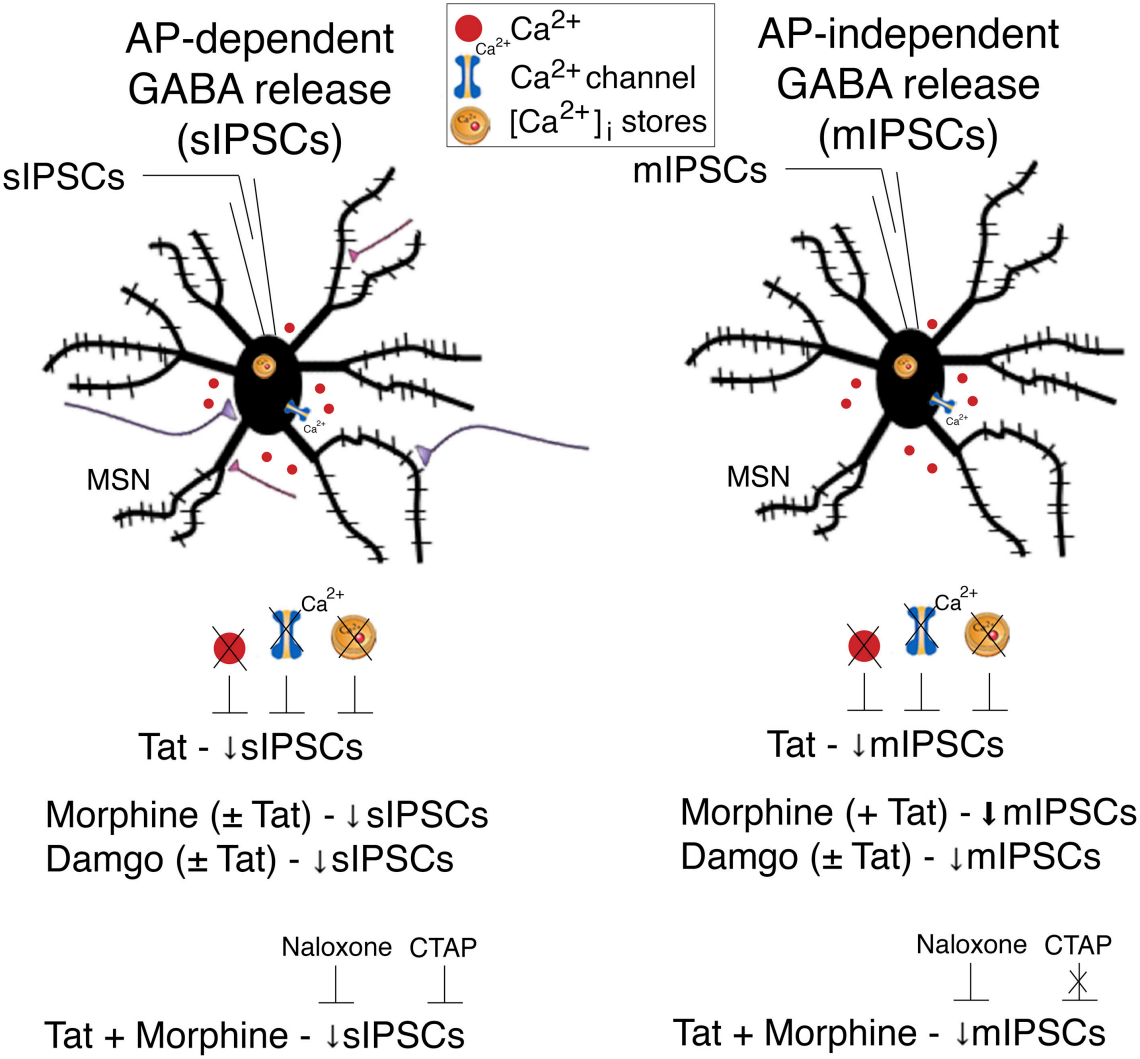
**Summary of the effects on inhibitory GABAergic neurotransmission by HIV-1 Tat and opioids**. Spontaneous inhibitory postsynaptic currents (sIPSCs) and miniature IPSCs (mIPSCs) were recorded from medium spiny neurons (MSNs) located in the dorsolateral striatum. A significant decrease in sIPSC frequency and mIPSC frequency was noted for Tat (10 nM) treatment. The significant Tat (10 nM) effect on the frequency of IPSCs was abolished when removing extracellular calcium (0 Ca^2+^), blocking voltage-dependent Ca^2+^ channels with cadmium chloride (CdCl_2_), and depleting intracellular endoplasmic reticulum calcium stores with thapsigargin, indicating that extracellular and intracellular calcium are necessary for the Tat-induced decrease in GABA release. For opioids, including morphine and damgo, separately and in combination with Tat, a significant decrease was noted for sIPSC frequency and mIPSC frequency, that was only potentiated for mIPSCs when combining Tat with morphine, but not damgo. Additionally, pretreatment with naloxone or CTAP prevented the combined Tat and morphine-induced decrease in sIPSCs frequency, whereas only naloxone, but not CTAP, prevented the combined Tat and morphine effect on mIPSCs frequency. It is hypothesized that the decrease of GABA release by Tat and opioids is primarily mediated via μ-opioid receptors with additive effects on GABA release being potentially mediated via additional opioid receptors. AP, action potential; MSN, medium spiny neuron; Ca^2+^, calcium; [Ca^2+^]_i_, intracellular calcium; sIPSC, spontaneous inhibitory postsynaptic currents; mIPSC, miniature inhibitory postsynaptic currents.

A summary of the findings of the present study is outlined in Figure 5. Our findings indicate that Tat as well as opioids, including morphine and damgo, separately, decreased AP-dependent and AP-independent GABA release in the striatum via a μ-opioid receptor-related mechanism, with Tat effects being dependent on extracellular and intracellular calcium. Combined Tat and opioid treatment revealed additive effects on GABA release only for Tat combined with morphine but not with damgo, which was blocked by naloxone but not CTAP. Based on the findings of the present study the decrease of GABA release by Tat and opioids is primarily mediated via μ-opioid receptors with additive effects on GABA release being potentially mediated via additional opioid receptors. Importantly, further studies are necessary to examine the effects of chronic Tat and opioid exposure on GABAergic neurotransmission to guide future research on HAND in the context of opiate drug use.

## Ethics statement

All applicable international, national, and/or institutional guidelines for the care and use of animals were followed.

## Author contributions

CX and SF designed the research; CX performed the electrophysiology experiments; CX and SF analyzed the data; CX and SF interpreted the data; SF wrote the paper; CX and SF discussed and edited the paper.

### Conflict of interest statement

The authors declare that the research was conducted in the absence of any commercial or financial relationships that could be construed as a potential conflict of interest.
